# Efficacy of phytase and/or chromium tripicolinate supplementation on dry matter and nitrogen digestibility and blood metabolites in grower pigs

**DOI:** 10.1093/jas/skae336

**Published:** 2024-11-02

**Authors:** Michael S Edmonds, Jon R Bergstrom, Thomas E Weber

**Affiliations:** Nutrition and Product Development, Kent Nutrition Group, Inc., Muscatine, IA 52761, USA; dsm-firmenich, Plainsboro, NJ 08536, USA; Innovative Solutions, Kent Nutrition Group, Inc., Muscatine, IA 52761, USA

**Keywords:** chromium, glucose, nutrient digestibility, phytase, pig

## Abstract

Phytase supplementation is widely used throughout the world for enhancing nutrient use efficiencies in pigs, while added chromium has been shown to help stabilize glucose metabolism by enhancing insulin sensitivity. Therefore, the objectives of this metabolism study were to examine the potential synergies of these additives to see if nutrient digestibilities and/or blood metabolites could be improved in grower pigs. A total of 12 Genesus terminal genetics grower pigs (20.7 kg) were allotted randomly in a crossover experiment with 4 periods and 4 dietary treatments based on a 2 × 2 factorial design via 2 groups. This provided 12 replicates per dietary treatment. Treatment (Trt) 1 consisted of a control diet without phytase while Trt 2 had decreased levels of soybean meal, calcium (Ca) and phosphorus (P) with added phytase (1,500 phytase units (FYT)/kg, HiPhorius; dsm-firmenich, Plainsboro, NJ). The nutrient release values for amino acids, calcium and phosphorus were via standard recommendations from dsm-firmenich for the phytase. Treatment 3 consisted of the control diet without phytase with 200 parts per billion (ppb) of added chromium from chromium tripicolinate (Chromax, Kent Nutrition Group, Inc., Muscatine, IA) while Trt 4 consisted of the diets with decreased levels of soybean meal, Ca and P with added HiPhorius (1,500 FYT/kg) and Chromax (200 ppb). With 6 metabolism crates available, 4, 1-wk-long periods were utilized to evaluate each of the 4 treatments with each pig with 2 groups evaluated and pooled for data analysis. The pigs were allowed a 4-d acclimation period followed by a 3-d collection period with the experimental diets fed at 4% body weight each day. Water was administered to each pig at 2.5 times the amount of feed fed each day. On the last day of the collection period, blood samples were collected before the meal (fasting) and then 2 h after the meal (postprandial). There were no significant differences among treatments for both fasting and postprandial glucose and insulin levels. Added phytase resulted in a reduction (*P* < 0.05) in fasting blood urea nitrogen (N). Nitrogen digestibility and retention and dry matter (**DM**) digestibility were all improved (*P* < 0.01) with pigs fed supplemental phytase. Supplemental chromium was without effect on any of the N and DM digestibility measurements. These data suggest that supplemental phytase has positive effects on improving N and DM digestibilities.

## Teaser

Finding technologies to improve the digestibility of protein in growing–finishing pig diets, which in turn reduces the excretion of nutrients into our environment, is important in enhancing the efficiency and sustainability of swine production to feed a growing population. Supplementation with phytase, such as HiPhorius, is one such technology that was clearly efficacious in improving nitrogen and dry matter digestibilities in these metabolism studies, while added chromium was without effect.

## Introduction

Phytic acid is an antinutrient in plants and oilseeds that increases endogenous amino acid losses (Cowieson et al., 2009) and acts as the key storage form of phosphorus via the sugar *myo*-inositol. Utilizing phytase to break down phytic acid, and thus releasing phosphorus and *myo*-inositol, has shown improvements in amino acid accretion and digestibility (Cowieson et al., 2017; Schmeiser et al., 2017; Lu et al., 2019). *myo*-Inositol has been shown to increase insulin sensitivity and thus improve glucose metabolism (Concerto et al., 2023). Feeding pigs a high dose of phytase increased the expression of glucose transporter 2 in the small intestine (Lu et al., 2020) which may lead to increased glucose absorption.

A meta-analysis of 35 papers (He et al., 2023) involving different chromium sources in weaned and growing–finishing pigs revealed that supplemental chromium picolinate was found to be the most beneficial in enhancing growth and carcass leanness in growing–finishing pigs. In another work (Santos et al., 2021), the use of chromium propionate was without effect on performance and carcass leanness in growing–finishing pigs fed under adequate or restricted space allowance. In data reviewed by Weber (2023) on sow productivity, added chromium tripicolinate resulted in an increase of litter size by 0.80 pigs per litter. Further work by Cox et al. (1987) suggested that an enhancement of insulin activity from supplementing chromium tripicolinate may help increase ovulation rate. Other potential benefits of supplemental chromium include decreasing the severity of rheumatoid arthritis (Hassouna et al., 2022), increasing the cytokine interleukin-6 (Myers et al., 1995), and decreasing the pro-inflammatory cytokine tumor necrosis factor-alpha (Myers et al., 1997) in growing pigs challenged with *E. coli* lipopolysaccharide. In a review by Valente Júnior et al. (2021), one key mechanism of action associated with chromium supplementation is the facilitation of insulin binding to the receptors on cell membranes, thus increasing the uptake of glucose into cells. This additional energy is used to increase protein synthesis which can increase lean tissue accretion.

The above studies describe some beneficial responses and mechanisms of action associated with added phytase and chromium when each was fed to swine and evaluated independently under a variety of conditions. To our knowledge, there are not any studies that have evaluated both added phytase and added chromium, fed separately and in combination, to determine the effects on dry matter (**DM**) and nitrogen digestibility and blood metabolites in growing pigs. Our hypothesis was that the combination of both ingredients would improve DM and nitrogen digestibility as well as lower urea nitrogen and glucose blood levels compared to when each ingredient was fed separately.

## Materials and Methods

All research protocols followed guidelines stated in the Guide for the Care and Use of Agricultural Animals in Agricultural Research and Teaching (FASS, 2010).

### General procedures

A total of 12 barrows (initial body weight, 20.7 kg) were individually housed in 6 stainless-steel metabolic crates and randomly allotted to 4 treatments based on 2 separate groups of 6 pigs that were pooled for analysis. The crates were 1.47 m long by 0.56 m wide. After their initial allotment, they were provided a 4-d acclimation period followed by a 3-d collection period. During the acclimation and collection periods, pigs were fed the experimental diets (meal form) at a level of 4% BW divided into 2 meals (morning and evening) per day. The experimental diets were formulated to meet or exceed the nutritional requirements for growing pigs (NRC, 2012). Water was provided to each pig at a level 2.5 times (milliliter) the amount in grams of feed fed each day. The allotment procedure for this study is shown in Table 1, which illustrates how the 4 treatments were balanced over a 4-wk period using the 6 barrows within each group and the stainless-steel metabolic crates that were available.

**Table 1.** Experimental design with 6 crates and 4 treatments (Trt)

Crate number	1	2	3	4	5	6
Phase 1 (days 0 to 7)	Trt 1	Trt 2	Trt 3	Trt 4	Trt 1	Trt 2
Phase 2 (days 7 to 14)	Trt 2	Trt 3	Trt 4	Trt 1	Trt 2	Trt 3
Phase 3 (days 14 to 21)	Trt 3	Trt 4	Trt 1	Trt 2	Trt 3	Trt 4
Phase 4 (days 21 to 28)	Trt 4	Trt 1	Trt 2	Trt 3	Trt 4	Trt 1

### Animals

Genesus terminal genetics pigs from the Kent Nutrition Group’s Research Farm were used in this study.

### Protocol and design for study

Four treatments were evaluated in a 2 × 2 factorial design. Treatment 1 consisted of the control diet without phytase while treatment 2 had decreased levels of soybean meal, calcium (Ca) and phosphorus (P) with added phytase (1,500 phytase units (FYT)/kg, HiPhorius; dsm-firmenich). The nutrient release values for amino acids, calcium and phosphorus and STTD phosphorus were via standard recommendations from dsm-firmenich for the supplemental phytase level. These 2 diets are shown in Table 2. Treatment 3 consisted of the control diet without phytase with 200 parts per billion (ppb) of added chromium from chromium tripicolinate (Chromax, Innovative Solutions). Treatment 4 had decreased levels of soybean meal, Ca and P with added HiPhorius and chromium tripicolinate to provide 200 ppb added chromium. Phosphorus laboratory analysis for treatments 1 to 4, respectively, was 0.55%, 0.32%, 0.59%, and 0.37%. With the Ca laboratory analysis, the values were 0.54%, 0.37%, 0.71%, and 0.48%, respectively, for treatments 1 to 4. In regard to the crude protein levels, the laboratory values were 19.94%, 18.81%, 19.63%, and 18.63%, respectively, for treatments 1 to 4. The phytase laboratory analysis revealed phytase levels of <100, 2,514, <100, and 2,277 FYT/kg for treatments 1 to 4, respectively. With the chromium laboratory analysis, the values were 1,560, 231, 1,545, and 285 ppb for treatments 1 to 4, respectively. A detailed explanation on the chromium analysis is in the discussion (Table 3 for all analysis).

**Table 2.** Diet formulation and calculated nutrient composition

Ingredients, % (as-fed)	Control	Phytase^1^	Phytase + assumed nutrient release values^2^
Corn, 7.0% CP	67.002	68.830	68.830
Soybean meal, 46.85% CP	29.710	28.844	28.844
Calcium carbonate	0.978	0.814	0.814
Monocalcium 21% phosphorus	0.920	0.069	0.069
Salt	0.350	0.350	0.350
Vitamin/trace mineral premix^3^	0.300	0.300	0.300
l-Lysine HCl	0.400	0.400	0.400
l-Threonine	0.168	0.165	0.165
dl-Methionine	0.150	0.145	0.145
l-Tryptophan	0.022	0.022	0.022
Calculated composition			
ME, Kcal/kg	3,326	3,361	—
NE, Kcal/kg	2,208	2,237	—
Crude protein, %	19.35	19.07	—
SID Lys, %	1.227	1.207	1.227
SID SAA, %	0.662	0.652	0.662
SID SAA:Lys	0.54	0.54	0.54
SID Thr, %	0.773	0.760	0.780
SID Thr:Lys	0.63	0.63	0.63
SID Trp, %	0.220	0.217	0.222
SID Trp:Lys	0.18	0.18	0.18
Total Ca, %^4^	0.66	0.45	—
Total P, %	0.56	0.38	—
Total Ca/total P (ratio)	1.18	1.18	—
Total STTD P, %	0.32	0.16	0.34

^1^Calculated added levels of 1,500 FYT/kg

^2^dsm-firmenich recommendations.

^3^The vitamin and trace mineral premix provided per kilogram of complete diet: 4,608 IU vitamin A, 1,152 IU vitamin D3, 21.84 IU vitamin E, 1.68 mg menadione, 0.0137 mg vitamin B12, 3.48 mg riboflavin, 12.36 mg d-pantothenic acid, 43.92 mg niacin, 42 mg Cu (copper sulfate), 60 mg Fe (ferrous sulfate), 0.66 mg I (calcium iodate), 13.68 mg Mn (manganese sulfate), 0.29 mg Se (sodium selenite), 60 mg Zn (zinc sulfate), and 47 mg Zn (zinc oxide).

^4^Total calcium was 0.45% based on phosphorus of 0.38% × 1.18 (ratio of Ca:P).

**Table 3.** Calculated and analyzed nutrients for treatments

	Treatments			
Item	1	2	3	4
Phytase, FYT/kg		1,500		1,500
Chromium tripicolinate, ppb			200	200
*Nutrients*				
Phytase analyzed, FYT/kg	<100	2,514	<100	2,277
Crude protein calculated	19.35	19.07	19.35	19.07
Crude protein analyzed, %	19.94	18.81	19.63	18.63
Ca calculated, %	0.66	0.45	0.66	0.45
Ca analyzed, %	0.54	0.37	0.71	0.48
P calculated, %	0.56	0.38	0.56	0.38
P analyzed, %	0.55	0.32	0.59	0.37
Chromium analyzed, ppb	1,560	231	1,545	285

### Sample collection and laboratory analysis

During the 3-d collection period, urine and feces from each pig were pooled within each pig each day. Total urine was collected into containers containing 40 mL of 2 normal hydrochloric acid to prevent nitrogen loss. The total volume of urine was recorded with water added to a set volume (i.e., 3,000 mL) and then a 100-mL aliquot was obtained each day and pooled and stored at −20 °C. Fecal samples were collected daily and pooled and stored at −20 °C. At the end of each collection period, fecal samples were dried for 96 h at 60 °C with the weights recorded and stored at −20 °C. Fecal samples were analyzed for DM and nitrogen (N) and urine samples for N at the Experiment Station Chemical Laboratories at the University of Missouri. Feed samples were analyzed for DM, minerals and N at the Eurofins Nutrition Analysis Center in Des Moines, Iowa. Phytase analysis was conducted by the dsm-firmenich Analytical Laboratory in Belvidere, NJ.

Fasting blood samples were obtained on the last day of the 3-d collections and then 2 h after feeding the pigs the morning meal. We obtained 6 to 8 mL of blood from the vena cava and placed in heparinized tubes and stored it on ice until centrifuging that afternoon. Samples were pipetted for plasma and stored at −20 °C until being shipped overnight with dry ice to the Department of Animal Sciences at Purdue University for analysis.

### Statistical analysis

Individual pigs served as the experimental unit within each phase for all statistical analyses. The data were analyzed as a randomized complete block design using ANOVA from Statistix 8 (Analytical Software, 2003; Tallahassee, FL). The experiment utilized a 2 × 2 factorial design consisting of added phytase (0 or 1,500 FYT/kg) and chromium (0 or 200 ppb). The crate served as a block and was included in the model. Differences were considered significant at *P* ≤ 0.05 and a tendency at *P* ≤ 0.10.

## Results

There were no significant differences between treatments for both fasting and postprandial glucose levels (Table 4). Glucose levels were about 10 to 18 units higher after the meal compared to the fasting levels. While there were no statistical differences, we did observe that the numerically lowest glucose levels were with the combination of phytase and chromium compared to either supplemented alone. Both fasting and postprandial insulin levels were also not significantly affected by treatment. However, there clearly were increased insulin levels postprandial of about 7 times the levels observed in the fasting stage. In regard to blood urea N, we did see a significant reduction in the fasting stage from supplemental phytase. Moreover, there was a trend (*P* = 0.08) for an interaction in which added phytase resulted in a greater reduction in urea N in the absence of chromium as opposed to when chromium was supplemented in the diet. In the postprandial stage, supplemental phytase tended (*P* = 0.08) to reduce blood urea N by 0.79 units or 11.65%. There were no significant differences for fasting and postprandial inositol levels from any of the treatments.

**Table 4.** Supplemental phytase and/or chromium tripicolinate (Cr) on serum parameters^1^

	Treatments							
Item	1	2	3	4				
Phytase (Ph), FYT/kg		1,500		1,500				
Cr, ppb			200	200	*P =*			
					Ph	Cr	Ph × Cr	SEM
Glucose fasting, mg/dL	113.61	112.33	112.49	108.48	0.60	0.62	0.78	4.93
Glucose fed, mg/dL	123.10	124.53	130.16	120.18	0.34	0.76	0.20	4.40
Insulin fasting, mU/L	1.64	1.93	1.77	0.97	0.70	0.54	0.42	0.66
Insulin fed, mU/L	11.47	8.96	12.28	12.75	0.65	0.32	0.51	2.26
Blood urea nitrogen fasting, mg/dL	6.63	4.82	6.21	6.05	0.04	0.38	0.08	0.46
Blood urea nitrogen fed, mg/dL	6.87	5.87	6.69	6.11	0.08	0.94	0.63	0.43
Inositol fasting, mg/dL	19.23	8.63	16.34	18.54	0.46	0.54	0.26	5.61
Inositol fed, mg/dL	16.80	15.10	19.00	13.43	0.45	0.96	0.69	4.81

^1^There were 12 individual pigs per treatment.

The fecal output was significantly less (*P* < 0.05) for pigs on supplemental phytase as opposed to those pigs not fed diets with the added phytase (Table 5). In addition, fecal N output was significantly reduced (*P* < 0.05) while urinary N output tended (*P* = 0.07) to be reduced for pigs on the diets with added phytase as opposed to those without the phytase. This in turn resulted in a significant reduction (*P* < 0.05) in total N output for pigs fed diets with supplemental phytase compared to those without the added phytase. Nitrogen digestibility was significantly improved (*P* < 0.05) with pigs fed diets containing phytase having an 85.30% digestibility compared to those pigs without the added phytase diets having a digestibility of 83.77%. Moreover, with N retention the average was 61.76% with supplemental phytase in the diets vs. 57.65% with diets devoid of the phytase. Furthermore, the pigs fed diets with supplemental phytase had significantly higher (*P* < 0.05; 87.22% vs. 85.61%) DM digestibility compared to those without the added phytase in the diets.

**Table 5.** Supplemental phytase and/or chromium tripicolinate (Cr) on nitrogen (N) and DM digestibilites^1^

	Treatments							
Item	1	2	3	4				
Phytase (Ph), FYT/kg		1,500		1,500				
Cr, ppb			200	200	*P =*			
					Ph	Cr	Ph × Cr	SEM
Feed intake, g	2,931	2,800	2,881	3,031	0.95	0.53	0.33	142
Fecal output, g	362.1	304.7	357.2	340.2	0.02	0.32	0.20	15.3
N intake, g	93.51	84.25	90.49	90.34	0.29	0.73	0.31	4.40
Fecal N output, g	14.63	12.32	14.95	13.04	0.001	0.32	0.70	0.52
Urine N output, g	24.65	20.45	23.68	21.18	0.07	0.94	0.64	1.79
Total N output, g	39.28	32.77	38.62	34.22	0.02	0.85	0.62	2.11
N digestibility, %	84.27	85.11	83.26	85.48	0.01	0.57	0.23	0.56
N retained, g	54.23	51.49	51.87	56.13	0.79	0.68	0.21	2.77
N retention, %	57.92	61.36	57.38	62.15	0.001	0.91	0.57	1.15
DM intake, g	2,528	2,418	2,493	2,659	0.82	0.41	0.27	123
DM digestibility, %	85.65	87.27	85.57	87.17	0.001	0.79	0.98	0.34

^1^There were 12 individual pigs per treatment.

## Discussion

In order to maximize the efficiency of the phytase at 1,500 FYT/kg, it is important to acknowledge the ability of phytase to improve the digestibility of P, Ca, and amino acids; so both the Ca and P were reduced in the diets formulated with phytase and a total Ca to total P ratio of 1.18 was applied to all diets as shown in Table 2. In previous research, ratios of total calcium to total phosphorus that went from 1.5:1 to 1.3:1 to 1.0:1 resulted in improved performance and bone mineralization in growing–finishing pigs fed diets with 500 phytase FYT/kg (Liu et al., 1998). The adverse effects reported with wider Ca/P ratios could be due to reduced phytase efficacy (Qian et al., 1996), and the formation of an insoluble phytate complex that prevents the hydrolysis by phytate (Pontoppidan et al., 2007; Selle et al., 2009). Moreover, in more recent research (Zhai et al., 2022, 2023), increased Ca to P ratios have impaired feed efficiency with phytase supplementation, but increased the deposition of Ca and P in bone as nursery pigs maintain a systematic balance between Ca and P excretions in urine.

In our study, supplemental phytase resulted in significant improvements in N digestibility (85.30% vs. 83.77%) as well as significantly better N retention (61.76% vs. 57.65%). Additionally, we observed a trend (*P* = 0.08) for a reduction in blood urea N by 0.79 units or 11.65% when phytase was added at 1,500 FYT/kg. These data agree with a review of 28 peer-reviewed papers by Cowieson et al. (2017) in which added phytase increased apparent ileal amino acid digestibility coefficients by 2.8% in grower pigs that averaged about 30 kg. In contrast, blood urea levels were not lowered from added phytase levels of 1,000 and 3,000 FYT/kg in grower (26 kg) pigs (Lu et al., 2019). In research with 18-d old broilers, the total tract excretion of N was reduced by 12% from 2 sources of added phytase (Zhang et al., 2022). These researchers interpreted the data as being a reduction in the antinutritional effects of phytic acid on endogenous amino acid flow (Cowieson and Ravindran, 2007) and a phytase-generated increase in ileal amino acid digestibility (Cowieson et al., 2017). In a review by Cowieson et al. (2009) in poultry, it was concluded that the improvements in amino acid digestibility from added phytase may be related to enhanced absorption of exogenous and re-absorption of endogenous amino acids.

In regard to the extra-phosphoric effects of super dosing phytase, Lu et al. (2019) observed 37% and 97% increases in plasma *myo*-inositol from supplementing 1,000 and 3,000 FYT/kg of phytase for 25 d in non-fasting grower (26 kg) pigs. These data are in contrast to our blood data in non-fasted pigs in which we found no changes in *myo*-inositol after only 7 d with added phytase at 1,500 FYT/kg. With broilers fed for 5 wk, supplemental phytase at 1,000 FTU/kg in a moderately P-deficient diet did increase plasma *myo*-inositol (Schmeisser et al., 2017). It appears from the above studies, that a longer time period may have been needed to show an increase in *myo*-inositol as we used 7 d rather than 25 to 35 d. In other studies (Rosso et al., 2023) using in vitro models to simulate the gastrointestinal tract of broilers, the addition of phytase at 2,000 FYT/kg increased *myo*-inositol release. With serum glucose, we did not observe any changes from added phytase which agrees with the data of Lu et al. (2019) in which they saw no differences in plasma glucose after 25 d in non-fasted pigs from both levels (1,500 and 3,000 FYT/kg) of added phytase.

In our study, we did observe a significant improvement (87.22 vs. 85.61) in DM digestibility from added phytase at 1,500 FYT/kg. These data are in contrast to Lu et al. (2019) in which they found no benefit on apparent ileal DM digestibility from supplementing diets with 1,000 and 3,000 FYT/kg of phytase in grower pigs. Further work in gestating and lactating sows (5-d adaptation followed by 5-d collection by grab sampling using titanium dioxide as an indigestible marker) with lower levels of phytase (187.5 and 375 FYT/kg) in diets devoid of inorganic phosphorus, also showed no improvement in apparent total tract digestibility of DM and crude protein (Zhai et al., 2021).

The addition of chromium tripicolinate was without effect on N digestibility, N retention, and DM digestibility in our research with pigs in a thermoneutral environment. This agrees with the work by Due et al. (2023), in which chromium picolinate did not improve N or DM digestibility in early finisher (average weight of 47 kg) pigs under both thermoneutral and heat-stressed environments. The heat-stress model was used since this type of stressor alters protein, lipid, and carbohydrate metabolism via modulating circulating insulin, yet no differences were observed. In a meta-analysis review of 35 papers (He et. al, 2023), supplemental chromium sources increased carcass leanness and loin eye area in growing–finishing pigs. However, in this extensive review there were no improvements in feed efficiency which would agree with our data in which added chromium did not improve N and energy digestibilities.

We observed no changes in fasting and postprandial glucose levels (2 h after the meal) nor urea N levels from supplemental chromium. This agrees with work by Lindemann et al. (2008), in which multiple chromium sources, fed at 25 times the recommended level for 75 d, had no effect in fasted finishing pigs for glucose and urea N levels. Furthermore, fasting glucose, urea N and insulin were also not significantly affected in pigs fed diets with added chromium (Amoikon et al., 1995; Matthews et al., 2001). In further work by Lindemann et al. (1995) with finishing pigs fed diets with supplemental (200 ppb) chromium ad libitum, there also were no changes in serum glucose and urea N levels. However, with gestating gilts, they did observe a numerical (*P* = 0.13) decrease in serum glucose from supplemental chromium 2 h post-fed compared to no changes in the fasting state. Lindemann et al. (1995) also found that there was a significantly smaller increase in insulin postprandial with added chromium as opposed to no added chromium which they stated as an improvement in insulin function from the added chromium. Our research clearly showed that insulin increased by over 7-fold when pigs were fed, as opposed to being fast, but we did not observe reduced insulin levels from added chromium.

Our research did not focus on glucose tolerance testing in which pigs would be sampled multiple times via catheters to see the effects of added chromium. Studies have shown (Amoikon et al., 1995; Guan et al., 2000) that significantly greater amounts of glucose are removed the first 20 min postprandial with dextrose solutions from various chromium sources with no significant differences observed in other research (Matthews et al., 2001). For our research, we obtained a single measurement 2-h postprandial to match up with the earlier work of Lindemann et al. (1995). In addition, we were evaluating whether an interaction could occur at this one time point with phytase combined with chromium since the breakdown of phytic acid via phytase along with chromium could have been synergistic with insulin sensitivity in terms of improving glucose utilization. Had this been a positive interaction (*P* = 0.20), in getting more glucose into cells postprandial, it could have had implications on enhancing pig performance with these 2 additives.

We observed no changes in fasting and postprandial insulin levels from added chromium as well as no changes in serum glucose after 7 d of feeding each treatment. In a review by Valente Júnior et al. (2021), they discuss the primary mechanism of action of chromium in potentiating the action of insulin by facilitating the binding of insulin to receptors on cell membranes. This in turn increases the translocation of a glucose transporter to the plasma membrane and thus improves glucose utilization by the cells. Perhaps the variation we observed in the statistical analysis with these blood measurements makes it hard to detect differences in insulin and glucose in our study as research by Salgado et al. (2022) showed variations in insulin sensitivity that was associated with de novo lipid synthesis in lean and fat finishing pigs. Work by Myers et al. (1997), in which pigs were exposed to an inflammatory challenge and had decreased glucose levels, also found that the supplementation of chromium was without effect. In humans with impaired glucose tolerance (Mertz, 1993), added chromium improved insulin efficiency and thus serum glucose levels.

Conducting an analysis of the test diets for chromium, when phytase is not added, is especially difficult due to the various inherent levels of chromium detected from ingredients such as monocalcium phosphate. We analyzed monocalcium phosphate and detected 110 parts per million (ppm) chromium which is adding 1,011 ppb of chromium to our 2 diets which had 0.92% in the formulas. When combined with the added limestone (7.18 ppm analyzed) and the other ingredients we had total chromium values of 1,560 (no chromium added) compared to 1,545 (with chromium added) ppb. A paper by Sullivan et al. (1994) clearly shows high levels of chromium (and iron) from monocalcium phosphate. And the 2012 NRC also states that the total chromium levels can range from 750 to 3,000 ppb in corn–soybean meal diets. Since 2012, the use of phytase has increased substantially in growing–finishing pig diets due to lower costs and greater efficacy of the evolving exogenous phytases. When we analyzed the diets with added phytase, which contained only 0.069% supplemental monocalcium phosphate and 0.814% limestone (both provided 134 ppb chromium to our diets), we found chromium levels of 231 and 285 ppb, respectively in the diets without and with added chromium. The point is that even when 200 ppb of chromium is added, it is especially difficult to show that increase in the test diets due to ingredient variation, especially from added monocalcium phosphate.

Finding technologies to improve the digestibility of protein in growing–finishing pig diets, which in turn reduces the excretion of N into our environment, is important in enhancing the efficiency and sustainability of swine production to feed a growing world. With added chromium, we observed no responses in N and DM digestibilities, nor were there any interactive effects with phytase. However, supplementation with a 4th-generation phytase, such as HiPhorius, is one such technology that was clearly efficacious in improving N and DM digestibilities in this metabolism study.

## Abbreviations

Ca: calcium
DM: dry matter
N: nitrogen
ppb: parts per billion
ppm: parts per million
P: phosphorus
FYT: phytase unit
